# HIT family genes: FHIT but not PKCI-1/HINT produces altered transcripts in colorectal cancer

**DOI:** 10.1038/sj.bjc.6690779

**Published:** 1999-11

**Authors:** J Elnatan, D Murphy, H-S Goh, D R Smith

**Affiliations:** Molecular Biology Laboratory, Tan Tock Seng Hospital, Moulmein Road, Singapore 308433, Republic of Singapore; Department of Medicine, University of Bristol, Bristol Royal Infirmary, Marlborough Street, Bristol BS2 8HW, UK

**Keywords:** colorectal, tumour, transcription, HIT genes

## Abstract

Forty-five colorectal adenocarcinomas were examined for alterations in the HIT family genes FHIT and PKCI-1/HINT by a combination of reverse transcriptase polymerase chain reaction and DNA sequencing. In all cases a single transcript corresponding to the reported sequence was detected using primers specific for the PKCI-1/HINT gene. In contrast multiple transcripts were detected using primers specific for the FHIT gene transcript. 6% (3/45) of tumours evinced no detectable expression of any FHIT transcript and a further 12% (6/45) produced only the normal full length transcripts. Ninety-six aberrant transcripts were characterized from the remaining tumours. Deviations from the normal full length sequence characterized included deletions, insertions of novel sequences, a point mutation as well as the usage of a putative alternate splice site in exon 10. Message variants were detected with approximately equal frequency in all tumour stages with the exception that templates with insertions were found solely in Dukes’ stage B tumours (*P* < 0.001). With the exception of the putative alternate splice site, aberrant transcripts were not detected in matched normal mucosa. These results suggest that members of the HIT family of genes are only selectively involved in tumorigenesis and that perturbation of FHIT gene expression is an early event in colorectal tumorigenesis. © 1999 Cancer Research Campaign


*British Journal of Cancer* (1999) **81**(5), 874–880
© 1999 Cancer Research Campaign
Article no. bjoc.1999.0779

### HIT family genes: FHIT but not PKCI-1/HINT produces altered transcripts in colorectal cancer

**J Elnatan[1], D Murphy[2], H-S Goh[1] and DR Smith[1]**

[1]Molecular Biology Laboratory, Tan Tock Seng Hospital, Moulmein Road, Singapore 308433, Republic of Singapore; [2]Department of Medicine, University of Bristol, Bristol Royal Infirmary, Marlborough Street, Bristol BS2 8HW, UK

**Summary** Forty-five colorectal adenocarcinomas were examined for alterations in the HIT family genes FHIT and PKCI-1/HINT by a combination of reverse transcriptase polymerase chain reaction and DNA sequencing. In all cases a single transcript corresponding to the reported sequence was detected using primers specific for the PKCI-1/HINT gene. In contrast multiple transcripts were detected using primers specific for the FHIT gene transcript. 6% (3/45) of tumours evinced no detectable expression of any FHIT transcript and a further 12% (6/45) produced only the normal full length transcripts. Ninety-six aberrant transcripts were characterized from the remaining tumours. Deviations from the normal full length sequence characterized included deletions, insertions of novel sequences, a point mutation as well as the usage of a putative alternate splice site in exon 10. Message variants were detected with approximately equal frequency in all tumour stages with the exception that templates with insertions were found solely in Dukes' stage B tumours ($P < 0.001$). With the exception of the putative alternate splice site, aberrant transcripts were not detected in matched normal mucosa. These results suggest that members of the HIT family of genes are only selectively involved in tumorigenesis and that perturbation of FHIT gene expression is an early event in colorectal tumorigenesis. © 1999 Cancer Research Campaign

**Keywords:** colorectal; tumour; transcription; HIT genes

The tumorigenic process leading to colorectal carcinoma formation involves multiple genetic alterations (Fearon and Vogelstein, 1990). Genes whose regulation or expression is known to be perturbed in colorectal carcinomas include tumour suppressor genes such as p53 and APC (Baker et al, 1989; Groden et al, 1991), proto-oncogenes such as *myc* and *ras* (Erisman et al, 1985; Bos et al, 1987), and genes of the mismatch repair system such as hMSH2 and hMLH1 (Fishel et al, 1993; Bronner et al, 1994). To date, however, no common series of genetic changes has been detected in all colorectal carcinomas, suggesting that tumorigenesis may result via a variety of pathways.

Most recently a novel putative tumour suppressor gene called FHIT (*F*ragile *H*istidine *T*riad) has been isolated and characterized (Ohta et al, 1996; Sozzi et al, 1996*a*). This gene resides at chromosomal location 3p14.2, an area known to undergo allelic loss in a variety of cancers, a phenomenon usually considered to be suggestive of the location of a tumour suppressor gene (Murphree and Benedict, 1984; Knudson, 1985). This gene spans the aphidicolin-inducible fragile site, FRA3B, a site first implicated in tumorigenesis in a family with renal cell carcinoma (Cohen et al, 1979; Wang and Perkins, 1984). Abnormalities of the FHIT locus were found in many established cell lines, and the gene was found to be abnormally transcribed in primary tumours of the digestive tract including oesophagus, stomach and colon (Ohta et al, 1996), the lung (Sozzi et al, 1996*a*) and breast (Negrini et al, 1996), with primary lesions affecting either overall RNA levels or specifically exons 5 and 8. These results suggest that FHIT could be abnormal in several common human tumour types. This is not, however, supported by one other group who have examined FHIT in a number of colorectal cancer cell lines and xenografts (Thiagalingam et al, 1996), and find a relatively low rate of abnormal FHIT expression.

The FHIT protein is a member of the histidine triad (HIT) family. FHIT's closest relatives are the yeast *Schizosaccharomyces pombe* diadenosine tetraphosphate hydrolase (*aph 1*) and a *Saccharomyces cerevisiae* putative *aph* gene (Huang et al, 1995). The HIT family represents an evolutionarily conserved group of small (14–22 kDa) proteins defined by two similarity blocks, one of which contains the H×H×H (HIT) motif. Interestingly, these homology blocks correspond to exons 5 and 8 of FHIT – both implicated in tumorigenesis. The first HIT protein to be described – protein kinase C (PKC) inhibitor-1 (PKCI-1) or HINT (histidine triad nucleotide-binding protein) – was isolated by Walsh from bovine brain (Pearson et al, 1990). Subsequently, human PKCI-1/HINT was identified in two separate yeast two hybrid screens, through interactions with either the regulatory region of PKCβ (Lima et al, 1996), or the ATDC gene product (Brzoska et al, 1995), which was isolated by complementation of the ionizing radiation sensitivity of ataxia telangiectasia (group D) cell lines (Kapp et al, 1992).

The role of FHIT aberrations in colorectal adenocarcinomas is in itself unclear. While altered FHIT transcripts have been identified in a range of tumour cell lines including those derived from the colonic adenocarcinomas (Ohta et al, 1996), other groups do not find a high frequency of altered transcripts (Thiagalingam et al, 1996), and so have suggested that FHIT may play little or no role in colorectal tumorigenesis.

Perhaps more confusingly while some groups detect aberrant transcripts only in the adenocarcinoma (Ohta et al, 1996) other

*Received 10 February 1999*
*Revised 27 April 1999*
*Accepted 29 April 1999*

*Correspondence to:* DR Smith groups have detected aberrant transcripts in normal colonic mucosae (Chen et al, 1997) similar to the situation found with other tumour types (Heubner et al, 1998). However, reports that show that transfection of wild-type FHIT into gastrointestinal tumour-derived cell lines (Siprashvili et al, 1997) is able to revert the tumorigeneic phenotype suggest that FHIT may well play a role in gastrointestinal and colorectal tumorigenesis. As such we undertook to define the status of FHIT and PKCI-1/HINT transcripts in a panel of primary colorectal adenocarcinomas.

### MATERIALS AND METHODS

### Patients and tumours

Samples consisted of 45 fresh, flash-frozen colorectal adenocarcinomas, each consisting of at least 70% neoplastic cells as verified histologically. All samples consisted of single adenocarcinomas, and patients with multiple carcinomas were excluded from the cohort. Tumours were staged Dukes' stage A–D by Turnbull's modification (Turnbull et al, 1967) of Dukes' original staging (Dukes, 1932).

### RNA extraction and first strand cDNA synthesis

Total RNA was extracted from tumour samples using Trizol® Reagent (Life Technologies, Gaithersburg, MD, USA). RNA was resuspended and solubilized in RNAase free water. Reverse transcription was performed in 20 μl final volume containing 2 μg total RNA, 30 ng random primers, 10 mM dithiotreitol (DTT), 0.5 mM dNTPs, 50 mM Tris–HCl (pH 8.3), 75 mM potassium chloride, 3 mM magnesium chloride and 200 units Superscript II (Life Technologies). The reaction was incubated for 10 min at 25°C, followed by 50 min at 42°C and terminated by heating at 90°C for 5 min. Integrity of all samples was verified by amplifying a 511 base pair fragment of the β-actin message using primers B1: 5′-GAAATCGTGCGTGACATTAA-3′ and B2: 5′-CTAGA-AGCATTTGCGGTGGA-3′.

### FHIT amplification and DNA sequencing

The primers used for the RT-PCR analysis of FHIT were as described by Ohta et al (1996). A total of 5 μl of first-strand cDNA reaction from above was incubated with 0.8 μM of primers 5U2 and 3D2 in a total volume of 50 μl also containing 50 mM of each dNTP, 5% dimethylsulphoxide, 0.5 mM spermidine and 2.5 U *Taq* DNA polymerase (Qiagen, Hilden, Germany) in 1 × polymerase chain reaction (PCR) buffer (Qiagen). PCR reactions involved an initial denaturation at 95°C for 5 min followed by 35 cycles of 1 min denature at 95°C, 1 min annealing at 62°C and 1 min extension at 72°C, followed by a final extension at 72°C for 10 min. A second nested PCR reaction was performed with 2 μl of first-round PCR reaction in total volume of 50 μl. The reagent conditions were as for first round amplification except that primers 5U1 and 3D1 replaced those used in the first-round amplification. PCR products were analysed on either a 1% agarose gel or a 1.8% Metaphor gel (FMC Rockland, ME, USA). The products were excised from the gel matrix and purified using the Qiaex PCR Purification Kit (Qiagen). DNA sequencing reactions were performed using the Big Dye Termination reaction kit (PE Applied Biosystems, Foster City, CA, USA) on approximately 60–90 ng of template from above. Analysis was undertaken on an automated ABI 377-18 DNA Sequencer (PE Applied Biosystems).

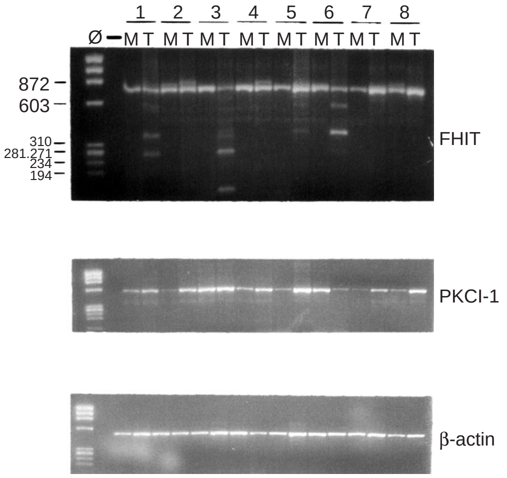

**Figure 1** RT-PCR analysis of transcripts expressed in eight colorectal tumours (T) and corresponding matching normal mucosa (M) from the same patients. Transcripts are shown for Fragile Histidine Triad (FHIT: top panel); protein kinase C inhibitor-1 (PKCI-1: middle panel) and β-actin (bottom panel). The position of the bands from Phi x174 DNA digested with H*ae*III are also shown. Truncated FHIT transcripts can be seen in tumour samples 1, 3 and 6

### PKCI-1/HINT amplification and DNA sequencing

A 550 base pair fragment of cDNA containing the entire coding sequence of the PKCI-1/HINT message was amplified from first-strand cDNA (as above) using 0.25 mM of primers : PKU : 5′-GAGAGAGGCCGAGATGGCAG-3′ and PKD : 5′-TAGCCATG-CAACAATGTCTT-3′ in a total reaction volume of 100 μl containing 20 μl of first-strand cDNA (from above), 0.2 mM of each dNTP, 1.5 mM magnesium chloride and 2.5 U *Taq* DNA polymerase (Promega, Madison, WI, USA) in 1 × PCR Buffer A (Promega). Reaction conditions included an initial denaturation of 5 min at 95°C followed by 30 cycles of 1 min denaturation at 95°C, 1 min annealing at 52°C and 1 min extension at 72°C, followed by a final extension of 10 min at 72°C. PCR products were analysed on 1% agarose gels, and the products excised from the gel matrix and purified using the Qiaex II purification kit (Qiagen). DNA sequencing was undertaken as above.

### RESULTS

We examined the message integrity of the HIT family genes FHIT and PKCI-1/HINT from 45 colorectal adenocarcinomas and their matched normal mucosa by a combination of reverse transcription PCR (RT-PCR) and DNA sequencing. Samples consisted of two Duke's stage A (tumour confined to muscularis propria); 13 Dukes' stage B (tumour penetrated through muscularis propria); 14 Dukes' stage C (involvement of regional lymph nodes) and 16 Dukes' stage D (evident distant metastasis) with staging according to Turnbull's modification (Turnbull et al, 1967) of Dukes' original staging (Dukes, 1932). All tumour samples contained at least 70% neoplastic cells as assessed histologically. Total RNA integrity was confirmed by amplification via RT-PCR of a 511 base pair fragment of the β-actin message (Figure 1).

© 1999 Cancer Research Campaign 

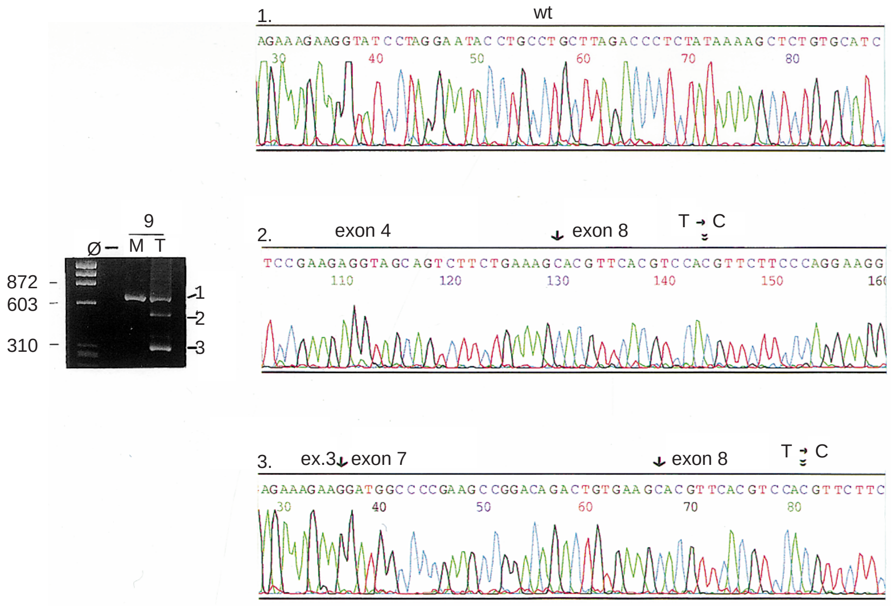

**Figure 2** Electrophoretic and sequence analysis of FHIT transcripts. Three products resolved by agarose gel electrophoresis after RT-PCR using FHIT specific primers were isolated and analysed by DNA sequencing using an automated DNA Sequencer. Products are normal full-length FHIT (*nflt*); transcript showing in-frame deletion of exons 5–7 (Δ*ex5–7*) and transcript showing in-frame deletion of exons 4–6 (Δ*ex4–6*). In the two truncated products the position of the His98 polymorphism is indicated. Expression of the polymorphic site in this sample is homozygous

**Table 1** Classes of FHIT transcripts in colorectal adenocarcinomas by stage

| Tumour stage | Wild-type | Insertions | Deletions | Mutations |
|---|---|---|---|---|
| A | nflt+Δex10/11b | Δex4–6+Δex10/11b | | |
| B | nflt | Δex4 | Δex4–6+Ins46b+Δex8+Δex10/11b | |
| | nflt+Δex10/11b | Δex4+Δex10/11b | Δex4–6+Ins14b | |
| | | Δex4–6+Δex10/11b | Δex4–7+Ins138b | |
| | | Δex4–8 | Δex4–7+Ins138b+Δex10/11b | |
| | | Δex4–8+Δex10/11b | Δex5–7+Ins93b+Δex10/11b | |
| | | Δex5–7+Δex10/11b | Δex6–8+Ins101b+Δex10/11b | |
| | | Δex5–8 | | |
| | | Δex5–8+Δex10/11b | | |
| | | Δex8 | | |
| C | nflt | Δex4 | | |
| | nflt+Δex10/11b | Δex4+Δex10/11b | | |
| | | Δex4–6 | | |
| | | Δex4–6+Δex10/11b | | |
| | | Δex4*–6+Δex10/11b | | |
| | | Δex4–8+Δex10/11b | | |
| | | Δex4*–10* | | |
| | | Δex5–7 | | |
| | | Δex5–7+Δex10/11b | | |
| | | Δex5–8+Δex10/11b | | |
| D | nftl | Δex4–6 | | nflt+pm37Q–L |
| | nflt+Δex10/11b | Δex4–7 | | |
| | | Δex4–7+Δex10/11b | | |
| | | Δex4–8+Δex10/11b | | |
| | | Δex5–6 | | |
| | | Δex5–6+Δex10/11b | | |
| | | Δex5–7 | | |
| | | Δex5–8+Δex10/11b | | |
| | | Δex8 | | |
| | | Δex8+Δex10/11b | | |

A total of 102 FHIT transcripts detected in 45 colorectal adenocarcinomas were fully characterized. Nftl: normal full-length transcript; Δ: deletion; ex: exon; pm: point mutation; Ins: insertion. Unless indicated by* deletions are in frame.

 *© 1999 Cancer Research Campaign*

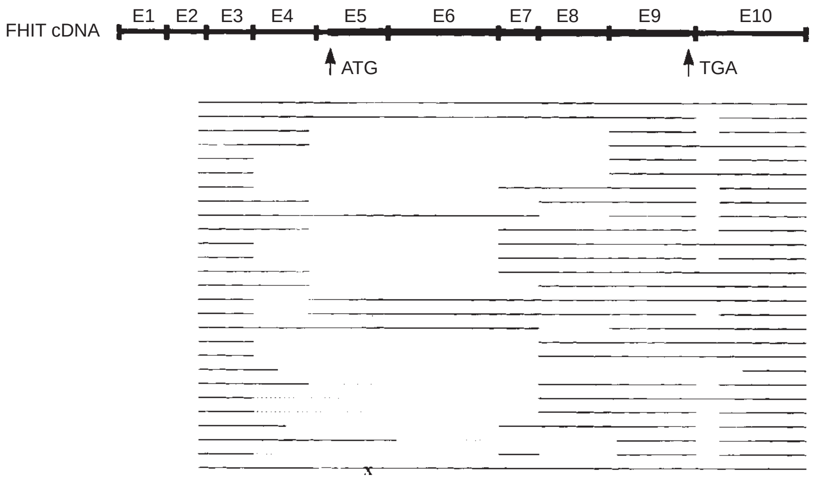

**Figure 3** Schematic representation of abberant FHIT transcripts. The classes of transcripts identified are shown in relation to the normal exonic structure of the FHIT transcript. Dotted lines: novel inserted sequences; x: mutation

Amplification of the message from PKCI-1/HINT was undertaken with primers which enabled amplification of the whole coding region. In each case only a single product corresponding to the expected size of the full-length product was detected in both the adenocarcinoma samples and the matched normal mucosa from the same patient (Figure 1). To detect any possible sequence alterations, each PCR product was fully sequenced on an automated ABI 377-18 DNA Sequencer. No sequence variations were seen in any product.

Amplification of messages of the FHIT gene was undertaken by a two-stage nested PCR strategy. Initial attempts to undertake a single-round amplification proved unsuccessful, probably due to low message levels (data not shown). The two-stage nested PCR methodology of Ohta et al (1996) was sufficient to identify multiple transcripts in tumour samples. Patterns of amplification were complex, with some tumours giving rise to as many as six separate products. In no case was a PCR product of less than full-length detected in any of the matched mucosa samples (Figure 1). Each product was initially identified through agarose gel electrophoresis and then the identity of the transcript determined by DNA sequencing as above (Figures 1 and 2). Classes of transcript detected are shown in Table 1 and Figure 3.

Three tumours gave no FHIT amplification product despite repeated attempts. These RNA samples, however, were used to successfully amplify both β-actin and PKCI-1/HINT messages, suggesting that these tumours have lost or had silenced both copies of the FHIT gene. These samples included two staged as Dukes' stage D and one staged as Dukes' stage B. Immunohistochemical examination of these three samples confirmed the absence of FHIT protein (data not shown).

A further six tumours gave only a single message corresponding to the normal, full-length transcript (termed *nflt*). Tumour stages were approximately equally represented, with the six tumours consisting of two tumours each from Dukes' stages B, C and D.

A total of 96 transcripts were characterized from the remaining 36 tumours (Table 1 and Figure 3). The most commonly found alteration was a deletion of eleven bases at the immediate start of the sequence coded by exon 10 (termed Δ*ex10/11b*). This deletion from the full-length sequence occurred in 65% (62/96) of non-full-length transcripts and 61% (62/102) of all transcripts. A further six tumours (one each of Dukes' stages A, B and C and three Dukes' stage D tumours) were found to only contain this message variant (*nflt+Δex10/11b*) and one tumour (Dukes' stage D) was found to contain only *nflt* and *nflt*+Δ*ex10/11b* transcripts. The Δ*ex10/11b* deletion was frequently found in transcripts with other alterations. This deletion was also detected in the matched mucosae from some patients (data not shown), and as such is consistent with the usage of an alternate internal splice site.

The remaining 29 tumours generated 88 transcripts. DNA sequence analysis determined that 42% (37/88) of these transcripts were either *nflt* or *nflt*+Δ*ex10/11*. The remaining 59% (51/88) of transcripts contained a variety of alterations including deletions (44 transcripts), deletion with insertions of novel sequences (six transcripts) and mutations (one transcript). Sixty-three per cent (32/51) of these transcripts also contained the Δ*ex10/11* deletion.

A total of 44 transcripts were defined with simple deletions, and with only two exceptions, deletions occurred in frame and resulted in the loss of between one and five exons in a single block. The two exceptions resulted in deletions from the middle of exon 4 (nucleotide 19 of exon 4) to the end of exon 6 and from the middle of exon 4 (nucleotide 54 of exon 4) to the middle of exon 10 (nucleotide 64 of exon 10; Figure 4). Of the 42 transcripts showing in-frame deletions 91% (39/43) would result in non-functional messages as they encompassed, to a greater or lesser degree, the start site for translation which occurs in exon 5, and included deletion of exons 5–8 (Δ*ex5–8*: eight transcripts), deletion of exons 4–6 (Δ*ex4–6*: seven transcripts), deletion of exons 4–8 (Δ*ex4–8*: six transcripts), deletion of exons 5–7 (Δ*ex5–7*: six transcripts), deletion of exons 4–7 (Δ*ex4–7*: two transcripts) and deletion of exons 5 and 6 (Δ*ex5–6*: two transcripts). The remaining deletion, which would result in a functional, or potentially functional, protein was the single deletion of exon 4 (Δ*ex4*: four transcripts), which occurred in one Dukes' stage B and one Dukes' stage C tumour.

Insertion of novel sequences occurred in six transcripts from five tumours. All of the insertions occurred in combination with in-frame deletions and included deletion of exons 4–6 with 46 base pairs of FHIT intron 4 sequences being transcribed in place of the deleted sequence (this transcript also had an in-frame deletion of exon 8 as well as the Δ*ex10/11b* variation : Δ*ex4–6+ins46+*Δ*ex8+*Δ*ex10/11b*);

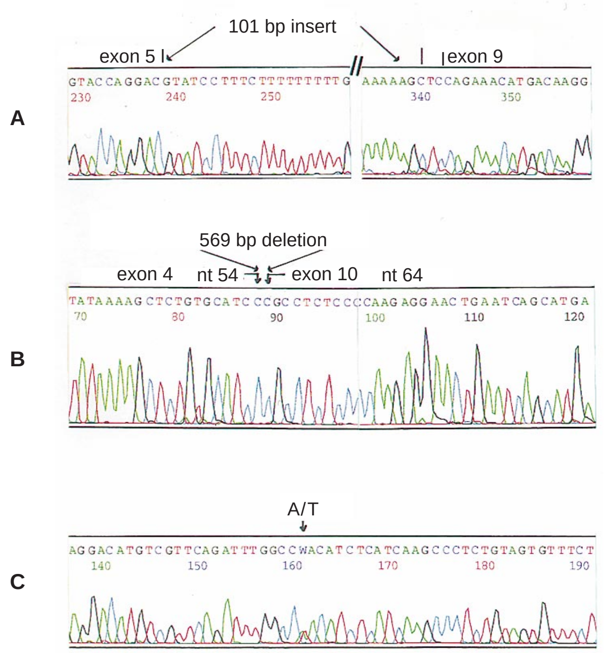

**Figure 4** Representative DNA electrophoretograms for transcript variants of FHIT. Depicted are a typical deletion/insertion aberrant transcript (**A**, Δ*ex6–8+Ins101b+*Δ*ex10/11b*), an atypical out of frame deletion transcript (**B**, Δ*ex4*–10**) as well as a rare non-sense mutation (**C**, nflt+pm37Q-L)

deletion of exons 6–8 with 101 base pairs of FHIT intron 4 sequences being transcribed in place of the deleted sequences (this transcript also transcribed the Δ*ex10/11b* variant: Δ*ex6–8+Ins101+*Δ*ex10/11b*; Figure 4); deletion of exons 4–7 with replacement of the deleted sequences with a 138 base sequence of unknown origin with high homology to Alu repeats (two transcripts, with the Δ*ex10/11b* variant in one: Δ*ex4–7+Ins138* and Δ*ex4–7+Ins138+*Δ*ex10/11*); deletion of exons 5–7 with replacement of the deleted sequences with 93 bases of an unknown sequence (this transcript also transcribed the Δ*ex10/11b* variant: Δ*ex5–7+Ins93+*Δ*ex10/11b*) and deletion of exons 4–6 with replacement of the deleted sequences with 14 bases of sequence of unknown origin (Δ*ex4–6+Ins14*).

Interestingly, all of the insertions occurred in Dukes' stage B tumours (Table 1) a distribution that is statistically significant (tumours with inserted transcripts, Dukes' stage B: 42% [5/13] > tumours with inserted transcripts, Dukes' stage A, C, D: 0% [0/33]; $P < 0.001$; Fisher's exact test).

One single-point mutation was detected, a CAA to CTA transversion at nucleotide 37 in exon 5 of tumour 2 (*nflt+pm37Q-L*), which would result in the replacement of a glutamine with a leucine (Figure 4). This tumour is Dukes' stage D, and it is clear that the mutation is present on only one of the two alleles and the second allele is present and being transcribed approximately equally.

Silent base changes were noted in 32 transcripts from 18 patients. The changes consisted of GCC to GCT at Ala88 (exon 7, nucleotide 15); GTC to GTT at Val97 (exon 8, nucleotide 12) and CAT to CAC at His98 (exon 8, nucleotide 15). Two of these changes (Ala88 and His98) have been previously identified as polymorphisms (Mao et al, 1996; Thiangalingam et al, 1996; Fong et al, 1997) and the third (Val97) was detected in the mucosae of selected patients again indicative of a polymorphism. The Ala88 polymorphism was detected in only one patient, while the His98 was detected in one-third (15/45) of all patients. Five patients carried the novel Val97 polymorphism. Three of these patients also had the His98 polymorphism. In contrast to others who have examined these polymorphisms in cell lines (Mao et al, 1996), these polymorphisms were frequently detected in a heterozygous pattern (data not shown), suggesting that both alleles of FHIT were present and actively transcribing, although homozygous expression was also seen (Figure 2).

### DISCUSSION

While FHIT and PKCI-1 are both members of the family of histidine triad proteins (HIT proteins), they are clearly differentially involved in tumorigenesis. Extensive analysis of PKCI-1 transcripts failed to find any alteration at the sequence level in a cohort of 45 colorectal adenocarcinomas and their matched normal mucosae. As such, the extensive preservation of sequence of the PKCI-1 gene may be suggestive of a vital housekeeping role within the cell.

In contrast, a range of altered transcripts were detected as being transcribed from the FHIT gene in the tumour samples. Alterations included deletion of exons (both in frame and out of frame), insertions of novel sequences and loss of transcripts entirely. At no time did we detect the production of any aberrant transcripts in the matched mucosae of the patients, supporting the contention that these transcripts are tumour specific (Ohta et al, 1996). Altered transcripts were detected approximately equally in all tumour stages suggesting that aberrations of FHIT gene transcription are a relatively early event in colorectal tumorigenesis.

Previous studies on the status of FHIT messages in colorectal adenocarcinomas have been contradictory (Ohta et al, 1996; Thiagalingam et al, 1996; Chen et al, 1997). In the original study by Ohta and colleagues (Ohta et al, 1996) aberrant FHIT transcripts were detected in three out of eight colonic tumours and analysis of matched normal tissues did not detect any alterations in the coding sequences. In contrast, Thiagalingam and colleagues detected only the normal full-length product in 27 of 31 colorectal cell lines and xenografts (Thiagalingam et al, 1996). Two samples gave no FHIT product and two samples showed evidence of full-length product together with smaller gene products, although these smaller products were not further analysed. More recently Chen and colleagues (Chen et al, 1997) analysed FHIT transcripts in 21 colorectal tumours. While they detected aberrant transcripts in ten tumours, they also detected aberrant transcripts in seven of the matched normal tissues.

The most significant difference between these studies lies in the use of nested PCR. While Ohta and colleagues (Ohta et al, 1996) use a two-stage amplification, as do Chen and colleagues (Chen et al, 1997), Thiagalingam and colleagues (Thiagalingam et al, 1996) use a single-step amplification and suggest that the high rates of aberrant transcripts seen by Ohta et al (1996) may be a result of over-representation of smaller products due to the nature of the nested PCR protocol. However, it should be noted that at no time have the studies that utilized a nested approach suggested that minor products are present at the same level as the full-length product. Indeed, Chen et al (1997) note that robust levels of the full-length product are present in 44 of the 46 tumours examined a result similar to that found in this study. As such, the question that needs to be addressed is a possible mechanistic action of the smaller transcripts in disrupting FHIT function.

 © 1999 Cancer Research Campaign

In this study a total of 86% of tumours examined contained altered or aberrant FHIT transcripts or expression, including three tumours that showed no FHIT transcripts at all. However, the most common transcript alteration was the deletion of 11 bases from the start of exon 10 (Δ*Ex10/11b*). This is after the translational stop codon located in exon 9, and so results in no functional change of protein coding. The sequence at which the deletion occurs: 5′-(deleted. … AG)AT … 3′ is identical to the sequence at the splice site between intron 9 and exon 10: 5′-(intron … ag) AT … 3′ and as such most probably represents the usage of an alternate splice site. Indeed this deletion was observed in normal colonic tissues in this study (data not shown) as well as in normal and tumourous tissues in other studies (Mao et al, 1996; Sozzi et al, 1996*a*; Hendricks et al, 1997). While it is clear from several gene systems that sequences in the 3′-untranslated region of a message can play a significant role in regulating message stability (Chew et al, 1994; Zehner et al, 1997; Russell et al, 1998) the presence of these spliced variants in the normal tissues of some patients makes it unlikely that this particular splice variant plays a role in tumorigenesis. If the Δex10/11b variant is considered an alternatively spliced message with no functional effect then normal FHIT transcripts are found in 26% (12/45) of tumours. One further tumour (tumour 45) contained only deletions of exon 4, again giving rise to a normal FHIT protein. In this case normal translation products would occur in 29% (13/45) of tumours.

Wild-type FHIT is able to suppress tumorigenesis in cell transfection studies (Siprashvili et al, 1997). In our analysis, however, only 8% (4/45) of tumours have lost the ability to produce functional FHIT transcripts. Two of these are Dukes' stage D, and two are Dukes' stage B. Loss of the ability to produce functional transcripts arises from complete loss or silencing of both alleles (three tumours) and deletion or insertions in all transcripts in a particular tumour (one tumour). The remaining 90% of tumours therefore have the ability to produce some functional FHIT protein. This is coupled with the observation that the majority of aberrant transcripts are apparently non-functional in that the whole of the FHIT coding sequence, or a substantial portion of it is deleted.

With only one exception all of the aberrant transcripts described here retain the sequences coded by exon 9, and it is perhaps of interest to note that aberrant FHIT transcripts from tumour types including ovarian, endometrial and cervical (Greenspan et al, 1997; Hendricks et al, 1997; Su et al, 1998), digestive tract (Chen et al, 1997), lung (Sozzi et al, 1996*a*; Fong et al, 1997), pancreatic (Simon et al, 1998), merkel cell carcinoma (Sozzi et al, 1996*b*), oesophageal (Michael et al, 1997), head and neck (Mao et al, 1996) and breast (Negrini et al, 1996) similarly retain exon 9 sequences in the aberrant transcripts. This may be particularly pertinent in the light of sequences present in this exon with high homology to the Kozaks translation initiation sequence (Kozak, 1984) and a small open reading frame, although as yet no peptide has been documented as arising from this site.

Point mutation of FHIT has been rarely described. Ahmadian and colleagues (Ahmadian et al, 1997) reported a G to T point mutation at nucleotide 651, which resulted in the substitution of a phenylalanine for the wild-type valine. Further analysis showed that this mutation was not tumour-specific but was in fact in the germline of the patient and moreover had been passed to one of the two daughters of the patient (Ahmadian et al, 1997). A single C to T mis-sense mutation at codon 61 resulting in a threonine to methionine mutation has been reported in a gastric signet ring cell adenocarcinoma has been reported by Gemma and colleagues (Gemma et al, 1997), although the nature of the amino acid substitution (uncharged polar to non-polar) leaves some doubt as to whether this is an inactivating mutation. The mutation detected here results in the substitution of a glutamine with a leucine. The fact that this substitution (uncharged polar to non-polar) is similar in nature to that detected by Gemma and colleagues (Gemma et al, 1997) may be of particular significance and suggests that examination of the function of point-mutated FHIT protein may shed important light on the normal function of FHIT. However, it must be noted that this mutation is located in neither the FHIT protein active site nor near surfaces known to be involved in nucleotide binding (Lima et al, 1997).

Three different polymorphic variants were detected in 18 patients. Two of these (Ala88 and His 98) have been well documented (Mao et al, 1996; Thiangalingam et al, 1996; Fong et al, 1997). The third variant was found at the Valine adjacent to the His98 polymorphism in exon 8, and was detected in five patients. Its detection in this cohort, which comprised solely Chinese colorectal cancer patients, may be indicative of a polymorphism of restricted penetrance. One further polymorphism has been reported by some authors (Fong et al, 1997) at Glu54 in exon 6, but this polymorphism was not detected in this cohort.

In our study, with the exception of the usage of the putative alternate splice site in exon 10, we detected no aberrant transcripts in the matched mucosae from the patients in this cohort. In this respect we are unable to confirm the results of Chen et al (1997), but rather support the observations of Ohta et al (1996) in that the production of aberrant transcripts in colorectal tumours is tumour-specific. The fact that the proportion of tumours with aberrant transcripts is not linked to tumour progression would also support a contention that inactivation of FHIT is a relatively early event in tumorigenesis. As such further investigations into the mechanism and consequence of FHIT gene action and inactivation are warranted.

### ACKNOWLEDGEMENTS

This work was supported by a grant from the Shaw Foundation and Grant RSCH/97/007 from Tan Tock Seng Hospital. DM thanks the Wellcome Trust for generous support.

### REFERENCES

Abderrahim H, Cohen D, Leppert M and White R (1991) Identification and characterisation of the familial adenomatous polyposis coli gene. *Cell* **66**: 589–600

Ahmadian M, Wistuba II, Fong KM, Behrens C, Kodagoda DR, Saboorian MH, Shay J, Tomlinson GE, Blum J, Minna JD and Gazdar AF (1997) Analysis of the FHIT gene and FRA3B region in sporadic breast cancer, preneoplastic lesions, and familial breast cancer probands. *Cancer Res* **57**: 3664–3668

Baker SJ, Fearon ER, Nigro JM, Hamilton SR, Preisinger AC, Jessup JM, vanTuinen P, Ledbetter DH, Barker DF, Nakamura Y, White R and Vogelstein B (1989) Chromosome 17 deletions and p53 gene mutations in colorectal carcinomas. *Science* **244**: 217–221

Bos JL, Fearon ER, Hamilton SR, Verlaan-de Vries M, van Boom JH, van der Eb AJ and Vogelstein B (1987) Prevalence of ras gene mutations in human colorectal cancers. *Nature* **327**: 293–297

Bronner CE, Baker SM, Morrison PT, Warren G, Smith LG, Lescoe MK, Kane M, Earabino C, Lipford J, Lindblom A, Tannergard P, Bollag RJ, Godwin, AR, Ward DC, Nordenskjold M, Fishel R, Kolodner R and Liskay RM (1994) Mutation in the DNA mismatch repair gene homologue hMLH1 is associated with hereditary non-polyposis colon cancer. *Nature* **368**: 258–261

Brzoska PM, Chen H, Zhu Y, Levin NA, Disatnik MH, Mochly-Rosen D, Murnan JP and Christman MF (1995) The product of the ataxia-telangiectasia group D complementing gene, ATDC, interacts with a protein kinase C substrate and inhibitor. *Proc Natl Acad Sci USA* **92**: 7824–7828

© 1999 Cancer Research Campaign

Chen YJ, Chen PH, Lee MD and Chang JG (1997) Aberrant FHIT transcripts in cancerous and corresponding non-cancerous lesions of the digestive tract. *Int J Cancer* **72**: 955–958

Chew LJ, Murphy D and Carter DA (1994) Alternatively polyadenylated vasoactive intestinal peptide mRNAs are differentially regulated at the level of stability. *Mol Endocrinol* **8**: 603–613

Cohen AJ, Li FP, Berg S, Marchetto DJ, Tsai S, Jacobs SC and Brown RS (1979) Hereditary renal-cell carcinoma associated with a chromosomal translocation. *N Engl J Med* **301**: 592–595

Dukes CE (1932) The classification of cancer of the rectum. *J Pathol Bacteriol* **35**: 323–332

Erisman MD, Rothberg PG, Diehl RE, Morse CC, Spandorfer JM and Astrin SM (1985) Deregulation of c-myc gene expression in human colon carcinoma is not accompanied by amplification or rearrangement of the gene. *Mol Cell Biol* **5**: 1969–1976

Fearon ER and Vogelstein B (1990) A genetic model for colorectal tumorigenesis. *Cell* **61**: 759–767

Fishel R, Lescoe MK, Rao MR, Copeland NG, Jenkins NA, Garber J, Kane M and Kolodner R (1993) The human mutator gene homolog MSH2 and its association with hereditary nonpolyposis colon cancer. *Cell* **75**: 1027–1038

Fong KM, Biesterveld EJ, Virmani A, Wistuba I, Sekido Y, Bader SA, Ahmadian M, Ong ST, Rassool FV, Zimmerman PV, Giaccone G, Gazdar AF and Minna JD (1997) FHIT and FRA3B 3p14.2 allele loss are common in lung cancer and preneoplastic bronchial lesions and are associated with cancer-related FHIT cDNA splicing aberrations. *Cancer Res* **57**: 2256–2267

Gemma A, Hagiwara K, Ke Y, Burke LM, Khan MA, Nagashima M, Bennett WP and Harris CC (1997) FHIT mutations in human primary gastric cancer. *Cancer Res* **57**: 1435–1437

Greenspan DL, Connolly DC, Wu R, Lei RY, Vogelstein JT, Kim YT, Mok JE, Munoz N, Bosch FX, Shah K and Cho KR (1997) Loss of FHIT expression in cervical carcinoma cell lines and primary tumors. *Cancer Res* **57**: 4692–4698

Groden J, Thliveris A, Samowitz W, Carlson M, Gelbert L, Albertsen H, Joslyn G, Stevens J, Spirio L, Robertson M, Sargeant L, Krapcho K, Wolff, E, Burt R, Hughes JP, Warrington J, McPherson J, Wasmuth J, Le Paslier D, Hendricks DT, Taylor R, Reed M and Birrer MJ (1997) FHIT gene expression in human ovarian, endometrial, and cervical cancer cell lines. *Cancer Res* **57**: 2112–2115

Huang Y, Garrison PN and Barnes L (1995) Cloning of the *Schizosaccharomyces pombe* gene encoding diadenosine 5′,5′′′-P1,P4-tetraphosphate (Ap4A) asymmetrical hydrolase: sequence similarity with the histidine triad (HIT) protein family. *Biochem J* **312**: 925–932

Huebner K, Garrison PN, Barnes LD and Croce CM (1998) The role of the FHIT/FRAB3B locus in cancer. *Ann Rev Genet* **32**: 7–31

Kapp LN, Painter RB, Yu LC, van Loon N, Richard CW, James MR, Cox DR and Murnane JP (1992) Cloning of a candidate gene for ataxia-telangiectasia group D. *Am J Hum Genet* **51**: 45–54

Kozak M (1984) Compilation and analysis of sequences upstream from the translational start site in eukaryotic mRNAs. *Nucleic Acids Res* **12**: 857–872

Knudson AG (1985) Hereditary cancer, oncogenes, and antioncogenes. *Cancer Res* **45**: 1437–1443

Lima CD, Klein MG, Weinstein IB and Hendrickson WA (1996) Three-dimensional structure of human protein kinase C interacting protein 1, a member of the HIT family of proteins. *Proc Natl Acad Sci USA* **93**: 5357–5362

Lima CD, D'Amico KL, Naday I, Rosenbaum G, Westbrook EM and Hendrickson WA (1997) MAD analysis of FHIT, a putative human tumour suppressor from the HIT protein family. *Structure* **5**: 763–764

Mao L, Fan YH, Lotan R and Hong WK (19???) Frequent abnormalities of FHIT, a candidate tumor suppressor gene, in head and neck cancer cell lines. *Cancer Res* **56**: 5128–5131

Michael D, Beer DG, Wilke CW, Miller DE and Glover TW (1997) Frequent deletions of FHIT and FRA3B in Barrett's metaplasia and esophageal adenocarcinomas. *Oncogene* **15**: 1653–1659

Murphree AL and Benedict WF (1984) Retinoblastoma: clues to human oncogenesis. *Science* **223**: 1028–1033

Negrini M, Monaco C, Vorechovsky I, Ohta M, Druck T, Baffa R, Huebner K and Croce CM (19???) The FHIT gene at 3p14.2 is abnormal in breast carcinomas. *Cancer Res* **56**: 3173–3179

Ohta M, Inoue H, Cotticelli MG, Kastury K, Baffa R, Palazzo J, Siprashvili Z, Mori M, McCue P, Druck T, Croce CM and Huebner K (1996) The FHIT gene, spanning the chromosome 3p14.2 fragile site and renal carcinoma-associated t(3;8) breakpoint, is abnormal in digestive tract cancers. *Cell* **84**: 587–597

Pearson JD, DeWald DB, Mathews WR, Mozier, NM, Zurcher-Neely HA, Heinrikson RL, Morris MA, McCubbin WD, McDonald JR, Fraser ED, Vogel HJ, Kay MC and Walsh MP (1990) Amino acid sequence and characterization of a protein inhibitor of protein kinase C. *J Biol Chem* **265**: 4583–4591

Russell JE, Morales J, Makeyev AV and Liebhaber SA (1998) Sequence divergence in the 3′ untranslated regions of human zeta- and alpha-globin mRNAs mediates a difference in their stabilities and contributes to efficient alpha-to-zeta gene development switching. *Mol Cell Biol* **18**: 2173–2183

Simon B, Bartsch D, Barth P, Prasnikar N, Munch K, Blum A, Arnold R and Goke B (1998) Frequent abnormalities of the putative tumor suppressor gene FHIT at 3p14.2 in pancreatic carcinoma cell lines. *Cancer Res* **58**: 1583–1587

Siprashvili Z, Sozzi G, Barnes LD, McCue P, Robinson AK, Eryomin V, Sard L, Tagliabue E, Greco A, Fusetti L, Schwartz G, Pierotti MA, Croce CM and Huebner K (1997) Replacement of Fhit in cancer cells suppresses tumorigenicity. *Proc Natl Acad Sci USA* **94**: 13771–13776

Sozzi G, Veronese ML, Negrini M, Baffa R, Cotticelli MG, Inoue H, Tornielli S, Pilotti S, De Gregorio L, Pastorino U, Pierotti MA, Ohta M, Huebner K and Croce CM (1996*a*) The FHIT gene 3p14.2 is abnormal in lung cancer. *Cell* **85**: 17–26

Sozzi G, Alder H, Tornielli S, Corletto V, Baffa R, Veronese ML, Negrini M, Pilotti S, Pierotti MA, Huebner K and Croce CM (1996*b*) Aberrant FHIT transcripts in Merkel cell carcinoma. *Cancer Res* **56**: 2472–2474

Su TH, Wang JC, Tseng HH, Chang CP, Chang TA, Wei HJ and Chang JG (1998) Analysis of FHIT transcripts in cervical and endometrial cancers. *Int J Cancer* **76**: 216–222

Thiagalingam S, Lisitsyn NA, Hamaguchi M, Wigler MH, Willson JK, Markowitz SD, Leach FS, Kinzler KW and Vogelstein B (1996) Evaluation of the FHIT gene in colorectal cancers. *Cancer Res* **56**: 2936–2939

Turnbull RB, Kyle K, Watson FR and Spratt J (1967) Cancer of the colon: the influence of the no-touch isolation technic on survival rates. *Ann Surg* **166**: 420–427

Wang N and Perkins KL (1984) Involvement of band 3p14 in t(3;8) hereditary renal carcinoma. *Cancer Genet Cytogenet* **11**: 479–481

Zehner ZE, Shepherd RK, Gabryszuk J, Fu TF, Al-Ali M and Holmes WM (1997) RNA–protein interactions within the 3′ untranslated region of vimentin mRNA. *Nucleic Acids Res* **25**: 3362–3370

 © 1999 Cancer Research Campaign
